# Solitary Plasmacytoma of the Mesentery: A Systematic Clinician's Diagnosis

**DOI:** 10.1155/2017/5901503

**Published:** 2017-05-11

**Authors:** Georgia Mitropoulou, Adamantia Zizi-Sermpetzoglou, Hippokrates Moschouris, Athanasios Kountourogiannis, Despoina Myoteri, Dionysios Dellaportas

**Affiliations:** ^1^Pathology Department, “Agia Sofia” Children's Hospital, Athens, Greece; ^2^Pathology Department, “Tzaneion” General Hospital, Piraeus, Greece; ^3^Radiology Department, “Tzaneion” General Hospital, Piraeus, Greece; ^4^2nd Department of Surgery, University Hospital “Aretaieion”, Athens, Greece

## Abstract

**Introduction:**

Plasmacytoma is an uncommon plasma cell neoplasm and its localized form is solitary plasmacytoma of the bone and solitary extramedullary plasmacytoma. Solitary plasmacytoma of the mesentery is extremely rare, reported only in a handful of cases.

**Case Presentation:**

A 47-year-old man with nonspecific abdominal complains was found to have an ill-defined mass on his mesenteric root. Laparoscopic biopsy and stepwise histopathological examination revealed a mesenteric plasmacytoma, and extensive imaging and laboratory investigations led to the diagnosis of the solitary mesenteric plasmacytoma. The patient underwent definitive radiotherapy and remains under remission one year later.

**Discussion:**

Plasma cell dyscrasias include a variant of proliferative disease, characterized by clonal expansion of bone marrow plasma cells, producing a massive quantity of monoclonal immunoglobulin called paraprotein or M-protein. Solitary extramedullary plasmacytoma accounts for only 3–5% of all plasma cell neoplasms. Meticulous adherence to the established diagnostic criteria helps the clinician to set the correct, yet very unusual and unexpected diagnosis.

## 1. Introduction

Plasma cell neoplasms result from clonal expansion of plasma cells which produce large amount of M-protein, with plasma cell myeloma being the most common disorder of plasma cell dyscrasias [1]. Plasmacytoma is an uncommon plasma cell neoplasm and its localized form is solitary plasmacytoma of the bone and solitary extramedullary plasmacytoma (SEP). The latter is extremely rare with a reported incidence less than 5% of plasma cell dyscrasias, affecting mostly the upper aerodigestive tract [2].

SEP of the mesentery has only been reported in a handful of cases, to the best of our knowledge [3]. A case of mesenteric plasmacytoma in a 47-year-old man is presented herein, initially managed as a pancreatic tumor, and the clinical and histopathological stepwise diagnosis is highlighted.

## 2. Case Presentation

**Figure 1:**
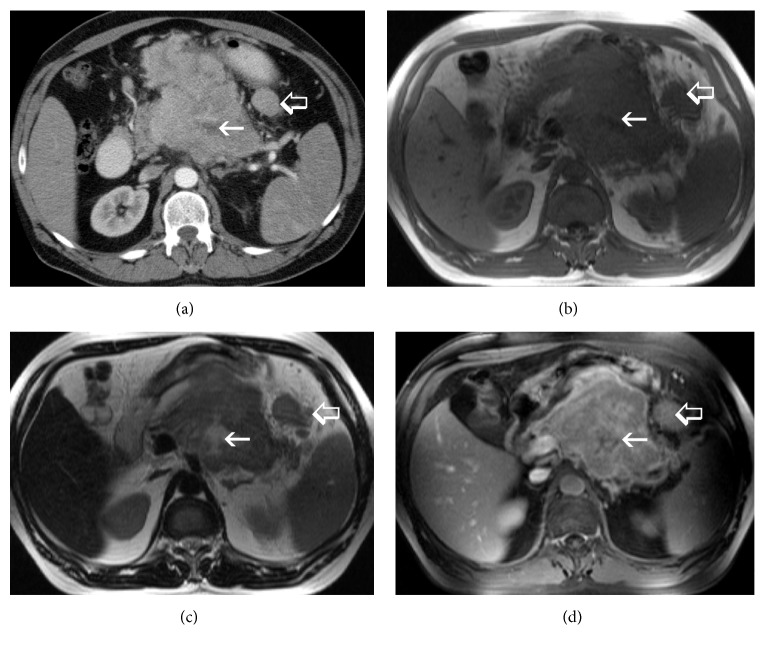
(a) Contrast-enhanced CT, (b) T1 weighted MR (c) T2 weighted MR, and (d) T1 weighted, contrast-enhanced MR. Arrows: the central part of the lesion lacks enhancement- necrotic part. Open arrows: satellite lesions are evident at the periphery of the mass.

**Figure 2:**
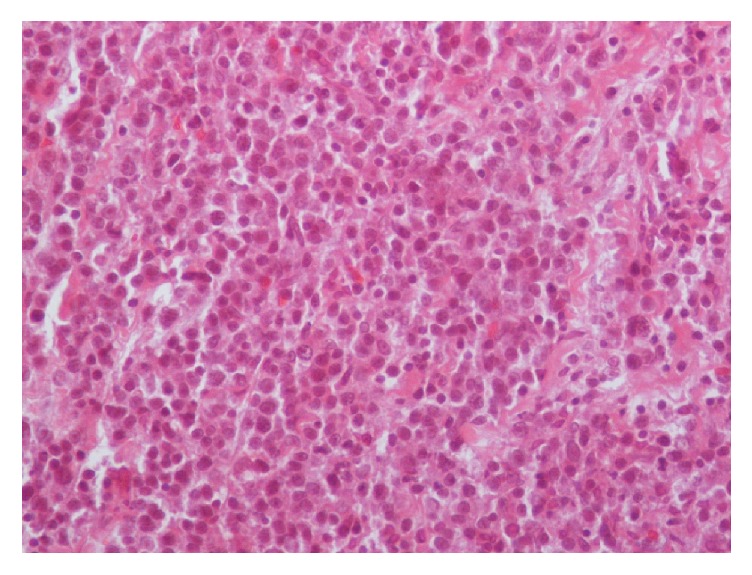
The neoplastic plasma cells showed variable degree of maturation with some of them being oval with round eccentric nucleus, “spoke wheel” chromatin, and no nucleoli, while others have prominent nucleoli or are occasional multinucleated (H-E ×200).

**Figure 3:**
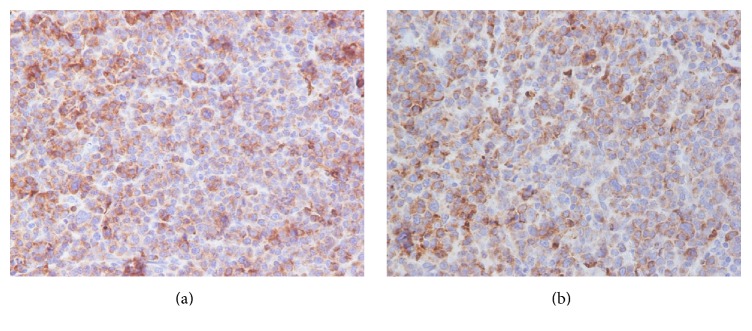
(a) The neoplastic cells were positive for CD138 (CD138 ×200). (b) The neoplastic cells were positive for CD38 (CD38 ×400).

**Figure 4:**
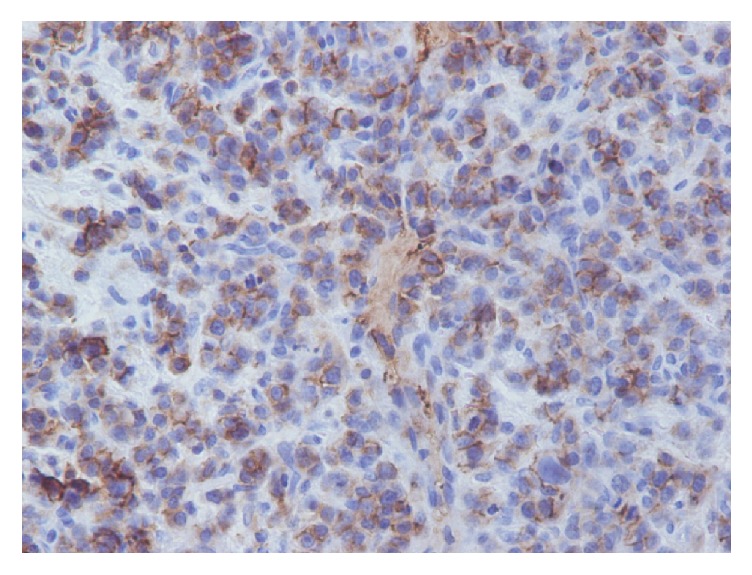
The neoplastic cells showed cytoplasmic monotypic IgA and *κ*-light chain restriction (*κ*  ×400).

A 47-year-old Caucasian male was admitted with nonspecific abdominal pain, vomiting, weight loss, and early satiety. The patient had no significant past medical history, and physical examination was inconclusive, apart from noting slight abdominal distension, with routine observations within normal limits. Laboratory investigations including complete blood count, biochemical and coagulation tests, and urinalysis examinations were unremarkable as well. Subsequently, a computed tomography (CT) of his abdomen was performed, revealing a multilobulated and ill-defined, soft tissue mass, arising from the root of the mesentery, compressing the posterior wall of the stomach (Figure 1(a)). To characterize the mass further an abdominal MRI was performed, showing peripheral enhancement and a likely necrotic center, with satellite lesions evident at the periphery of the mass (Figures 1(b), 1(c), and 1(d)). Radiological evaluation opted towards a lymph nodal mass of unknown origin. After multidisciplinary meeting team discussion (MDT) a laparoscopic biopsy was decided and performed, deemed to be the safest approach. Histopathological examination revealed a 3 × 1 × 0,4 cm solid, soft, and tan mass. On microscopic examination, the neoplasm was composed of neoplastic plasma cells showing variable degree of maturation. Some of the cells were oval with round eccentric nucleus, “spoke wheel” chromatin, and no nucleoli. Other cells appeared with prominent nucleoli and occasional multinucleated. Mitotic figures were also apparent (Figure 2). Immunohistochemically these cells were positive for CD138 (Figure 3(a)) and CD38 (Figure 3(b)) while CD56 was expressed in a small percentage of neoplastic cells. Other markers such CD20, CD79a, CD3, CK20, CK7, chromogranin, synaptophysin, and HHV8 were negative, whereas proliferative index MIB-1 was high. The neoplastic cells also showed cytoplasmic monotypic IgA and *κ*-light chain restriction (Figure 4).

All the above set the diagnosis of mesentery plasmacytoma, which was finally classified as SEP of the mesentery, since bone marrow examination was normal with less than 5% of plasma cells and concentrated urine was negative of Bence-Jones protein, while no other site of disease was revealed on additional imaging investigations, and renal parameters and calcium levels were normal. Moreover, serum protein electrophoresis did not show any monoclonal protein spike. The final diagnosis was IgA- *κ* type solitary extramedullary plasmacytoma of mesentery. The patient was treated with 50 Gy of radiotherapy, delivered over six weeks, and developed mild radiation related diarrhea. The tumor responded very well, with complete radiological response, and the patient remains disease free one year later, on follow-up visits.

## 3. Discussion

Plasma cell dyscrasias include a variant of proliferative disease, characterized by clonal expansion of plasma cells in the bone marrow or other tissues, producing a massive quantity of monoclonal immunoglobulin called paraprotein or M-protein [4]. The main disorders of this group are plasma cell myeloma, plasmacytoma, and conditions which result from immunoglobulin deposits in tissues. Plasma cell myeloma, characterized by disseminated proliferation of plasma cells, is a bone marrow origin neoplasm. In contrast plasmacytoma, which is quite rare, appears as a unique focus of neoplastic plasma cells, occurring either in bone (solitary plasmacytoma of bone) or in soft tissue (solitary extramedullary plasmacytoma). Immunoglobulin deposition diseases include primary amyloidosis and light and heavy chain deposition diseases [5].

Solitary extramedullary plasmacytoma (SEP) is a rare tumor accounting for only 3–5% of all plasma cell neoplasms [6]. It is defined as either primary, when it occurs as isolated mass, or secondary, when it is associated with generalized multiple myeloma. Although SEP can occur in almost any tissue, it mostly involves the upper aerodigestive tract [7]. Other sites of involvement include the gastrointestinal tract, breast, thyroid, skin, urinary bladder, lymph nodes, testis, and CNS [8]. It affects more frequently males than females in the sixth decade of life [1].

The clinical features are related to the anatomic site that is involved. The diagnosis of SEP is based upon the following recommended diagnostic criteria compiled by Soutar et al. [9]: (1) histopathological tissue evidence of single area of extramedullary involvement of monoclonal plasma cells; (2) normal marrow without clonal involvement; (3) normal results on skeletal imaging survey; (4) no anemia, hypercalcemia, or renal impairment, attributable to plasma cell dyscrasias; and (5) no or low serum or urinary levels of monoclonal immunoglobulins.

In the case reported above these criteria were fulfilled.

Serum protein electrophoresis should be performed whenever multiple myeloma or related disorders are suspected, and this can be used as a prognostic marker if positive at presentation [10].

On histopathological examination SEP is straightforward to diagnose, except for cases where the tumor cells are poorly differentiated. Microscopically, the neoplasm consists of plasma cells of variable degree of plasmacytic maturation. Mature cells usually have clumped nuclear chromatin, abundant cytoplasm, and no nucleoli. Immature cells often have large nuclei with prominent nucleoli. In some cases, multinucleated and pleomorphic plasma cells may be present. Amyloid may also be present in 10% of the cases. Immunohistochemical examination with antibodies to kappa and lambda light chains is essential to confirm the diagnosis. The immunophenotypic features are similar to those of plasma cell myeloma. The cells usually express CD138 and are strongly positive for CD38. The differential diagnosis of plasmacytoma includes plasma cell granuloma, plasmacytoid lymphoma, large cell lymphoma, and marginal zone lymphoma of MALT type [11]. Supplementary laboratory and radiologic examinations are useful in order to exclude remote disease sites [12].

SEP is considered radiosensitive and therefore the treatment of choice is radiation therapy [13]. Despite surgical resection is thought to be equally effective to radiotherapy, their combination is preferred for large masses that can be surgically resected safely [14].

The radiotherapy field in the case presented above was indeed large, and it was very challenging to manage the patient's postradiation enteritis. Radiotherapy delivered in fields involving the small bowel can cause significant short- and long-term side-effects, which can be equally troublesome to manage [15]. The optimal radiotherapy dose is not accurately defined, due to the rarity of the disease. Doses below 20 Gy were used as palliative and between 25 and 50 Gy were used for cutaneous lesions in a recent case series, with mixed results [13].

The effectiveness of adjuvant chemotherapy is not thoroughly investigated and is used for refractory or relapsed disease. However, chemotherapy is considered in the adjuvant setting for tumors larger than 5 cm and high-grade histology [16]. Regional recurrence and distant metastasis may develop in 22% of patients and 15% will develop generalized myeloma [8]. SEP prognosis is considered good with a median survival of approximately 10 years [6].

The follow-up has no established protocols or guidelines and it has to be mentioned that our patient's follow-up is short, compared to reported survival from SEP [13].

In conclusion, osseous and extraosseous plasmacytomas are localized tumors of monoclonal plasma cell origin. Although extramedullary plasmacytomas occur mainly in the upper aerodigestive tract, mesentery may also be involved with very few case reports in current literature. The sound combination of radiological, laboratory, and histopathological work-up leads to the correct diagnosis, and subsequent treatment is highly effective, for a disease with impressive and diverse initial presenting features.
